# Influence of the Chemical Composition of Ceria Conversion Coatings, Sealed in Solution of NaH_2_PO_4_ and Ca(NO_3_)_2_, on the Corrosion Behavior of Aluminum

**DOI:** 10.3390/ma16196499

**Published:** 2023-09-29

**Authors:** Aleksandar Tsanev, Reni Andreeva, Dimitar Stoychev

**Affiliations:** 1 Institute of General and Inorganic Chemistry, Bulgarian Academy of Sciences, “Acad. G. Bonchev” Street, bl. 11, 1113 Sofia, Bulgaria; 2 “Rostislaw Kaischew” Institute of Physical Chemistry, Bulgarian Academy of Sciences, “Acad. G. Bonchev” Street, bl. 11, 1113 Sofia, Bulgaria; randreeva@ipc.bas.bg (R.A.); stoychev@ipc.bas.bg (D.S.)

**Keywords:** aluminum, ceria, calcium, phosphate(s), conversion coatings, corrosion

## Abstract

The corrosion-protective influence of eco-friendly ceria conversion coatings deposited on Al-1050 alloy, additionally treated in mixed NaH_2_PO_4_ and Ca(NO_3_)_2_ solution, was studied. The main aim of this work was to investigate how the obtained mixed systems of coatings eliminates the negative role of cracks and pores on the surface formed after deposition only of ceria coating. For this purpose, the growth structure, main components and corrosion resistance of the so formed protective systems were investigated by SEM, EDS, XRD, XPS and electrochemical (PDP, Rp, etc.) methods. The results obtained show that the basic components of the conversion layers (before and after exposure in model corrosion media) are characterized by Al_2_O_3_, Al(OH)_3_, CePO_4_ and Ca_5_(PO_4_)_3_(OH). Based on these results, the optimal conditions of immersion treatment(s) of Al substrate are established. At these regimes, the relationship of co-deposited Ce^3+^, PO_4_^3+^ and Ca^2+^-containing components of the conversion layers determine the maximum values of their polarization resistance—a basic criterion for corrosion protection of Al. This effect is related to the formation of fill out of the defects of the conversion coatings and additional Ca_5_(PO_4_)_3_(OH), CePO_4_ AlPO_4_ and Al(OH)_3_ deposits, leading to the decrease of the corrosion rate.

## 1. Introduction

The requirements and norms of modern legislation [1,2], regarding the application of “green” technologies in the deposition of conversion protective and decorative coatings on aluminum and its alloys (Al), strictly exclude the widely used until recently highly efficient chromate conversion coatings based on Cr^6+^ [3,4,5,6,7,8]. A wide range of intensive research carried out in this aspect ranks among the favorites of ecofriendly technologies those related to the formation of protective oxide layers of lanthanides and, in particular, cerium oxides [9,10,11,12,13,14,15,16,17,18,19,20,21,22,23]. However, upon application of the cerium salts, elaborated on the basis of these compositions and regimens for ceria conversion treatment of Al, the high degree of protection of the chromate layers has not yet been achieved. In this connection, systematic investigations continue and they are associated with finding options for promoting their corrosion-protective ability by the inclusion of additional processing of the formed ceria conversion coatings (CCOC). Encouraging results in this respect have been obtained by the inclusion of additional finishing sealing of the already deposited CCOC in solutions of various phosphate compounds, leading to the formation of a thin phosphate layer [5,15,24,25,26,27,28]. Such an approach could lead to the formation of insoluble Ce-phosphates, as well as to a possible synergistic protective effect between ceria layers and the combined metal-phosphate layer formed on it, where the metal could be Ce, Al or Al + Ce [15,29].

It is necessary to note, however, one characteristic disadvantage of these conversion coatings, which is related to the formation of micro-cracked pores [9,30,31]. They can reach the Al substrates and cause corrosion damage when in contact with aggressive environments.

In light of the above, the task of the present study was to establish and juxtapose corrosion behavior, with respect to the efficiency of deposited-on-Al-1050 thin, conversion cerium-oxide coatings (CCOC) [32,33,34,35]. They are subjected to additional sealing post-treatment in mixed solution of sodium phosphate mono basic and calcium nitrate. Sodium phosphate mono basic and calcium nitrate can fill and reduce possible crack and pore defects of the CCOC, as well as increase their corrosion-protective influence and ability in corrosion medium.

Our motivation for including and investigating the impact of such additional processing on CCOC is the results of recent systematic studies of H.S. Lee and co-authors [36,37,38,39]. They found a strong positive effect of sodium phosphate and calcium nitrate sealing treatment on the corrosion resistance and ability of wire arc sprayed aluminum protective coatings (deposited on steel substrate), the surface of which is characterized by a rough and porous microstructure.

## 2. Materials and Methods

The studied conversion layers (produced by electroless treatments) were deposited on substrates of “technically pure” Al 1050 (containing 0.40% Fe, 0.25% Si, 0.05% Mn, 0.05% Cu, 0.07% Zn, 0.05% Mg). The studied specimens/substrates of dimensions 3 × 3 cm were cut out of rolled Al sheets (not polished additionally) with thickness 0.1 cm. Their pre-treatment (in 1.5 M NaOH) is described in [32]; The short name for these pre-treated substrates is S1. The formation of thin conversion ceria oxide coatings on S1 was implemented in a solution containing 0.5 M CeCl_3_ × 7H_2_O + 1 × 10^−5^ M CuCl_2_ × 2H_2_O (at a temperature 25 °C and with a time of immersion 2 h). No H_2_O_2_, or other type of oxidizing agent, was added [33,34,35]. For these systems, the short name is S2. The thickness of the deposited CCOCs was 110 nm (as measured from XPS in-depth profiles, displaying in detail the change of the ratio between the elements Ce, Al and O in the depth of CCOC [35]). After formation of CCOC, the specimens were rinsed fully in distilled water, dried and post-treated in mixed 0.5 M NaH_2_PO_4_ + 0.1 M Ca(NO_3_)_2_ solution, as proposed and applied for formation of phosphate protection layers (marked in the text below as PhCa) on aluminum by Lee and co-authors [36]. The temperature of the mixed solution was 85 °C and we chose three different times of immersion treatment: (I) 5 min; (II) 3 × 5 min and (III) 3 × 5 min + 72 h exposure of the so treated sample in humid atmosphere over a distillated water (in thermo-stated glass cell at 50 °C). In procedure (I), after 5 min immersion treatment, the sample(s) were rinsed fully in distilled water and finally dried at room temperature. In procedure (II), the sample(s) were under immersion treatment, rinsing and drying three times after every 5 min of immersion treatment in the mixed solution. In procedure (III), the sample(s) were under immersion treatment, rinsed and dried three times after every 5 min immersion treatment (as in procedure II), after which the sample(s) were left in a humidity chamber [37,38,39] for 72 h at 50 °C and 95% relative humidity atmosphere for formation of natural oxides and submitted to a final drying, prior to exposure and investigation in model corrosion medium of 0.1 M NaCl (CM). The thickness of the so deposited phosphate layers was ≈0.9 µm (measured by SEM observations of cross sections of the samples). The short names of these prepared samples: for sample (I)–S3; for sample (II)–S4 and for sample (III)–S5 (Table 1).

The surface morphology, structure and chemical composition in the bulk of the studied systems were investigated by electron microscopy (JEOL JSM 6390, Tokyo, Japan) under the conditions of secondary electron image (SEM), back-scattered electrons (BEI) and characteristic energy dispersive X-rays analysis (EDS).

The formed phases on the S1 substrate(s) covered by CCOC, after their post-treatment in NaH_2_PO_4_ and Ca(NO_3_)_2_ solutions, were observed by X-ray diffraction. XRD patterns were recorded on the multipurpose system Empyrean, manufactured by PANalytical, Malvern, UK. The system was equipped with LFHR X-ray tube Cu Kα radiation (λ = 1.54184 Å) generated at 45 kV and 40 mA. The volume fraction (in %) of each phase formed on the samples was analyzed by HighScorePlus 4.5 software and the ICSD database (Inorganic Crystal Structure Database) provided by FIZ Karlsruhe, Eggenstein-Leopoldshafen, Germany.

The X-ray photoelectron spectroscopy (XPS) was carried on an AXIS Supra electron-spectrometer (Kratos Analitycal Ltd., Manchester, UK) using achromatic AlKα radiation with photon energy of 1486.6 eV. The analyzed area was 0.3 × 0.75 mm^2^. The energy calibration was performed by fixing the C1s line of adsorbed adventitious hydrocarbons to 284.6 eV. The binding energies (BE) were measured with an accuracy of ±0.1 eV. The changes in composition and chemical surrounding of the chemical elements were determined monitoring the areas and binding energies of the photoelectron peaks of the appropriate elements, that is, C1s, O1s, Ce3d, etc. The chemical composition (in at. %) and the state of the elements of cerium-based CCOC on Al 1050 were examined and determined for the as-deposited samples and after their post-treatment in mixed 0.5 M NaH_2_PO_4_ + 0.1 M Ca(NO_3_)_2_ solution, as well as after long-time (up to 168 h) exposure in model corrosion media.

The corrosion behavior of the samples was studied in a standard three-electrode cell in open-to-air 0.1 M NaCl (“p.a.” Merck, Darmstadt, Germany) CM at 25 °C. A platinum electrode was used as the counter electrode, having dimensions of 10 × 10 × 0.6 mm, while the reference electrode was saturated calomel electrode (SCE) (E_SCE_ = +0.240 V vs. SHE). All the potentials in this study are compared to SCE (for the realization of OCP-Time plots, Chronoamperometry (at E_pitt_) and Rp measurements, the reference electrode was Ag/AgCl). The anodic and cathode polarization curves were obtained by means of a potentiostate/galvanostate Gamry Interface 1000, the obtained results being processed with the help of specialized software. The potentiodynamic polarization (PDP) curves were recorded at a sweeping rate of the potential 1 mV.s^−1^ within the range of potentials–2500–+2500 mV (starting in cathodic and anodic directions from the open circuit potential (E_OCP_) of S1 = −0.660 V vs. E_SCE_).

The polarization resistance investigations of studied samples (as-deposited and after definite time of exposure in CM) were carried out on Gamry Interface 1000 (Software and Frequency Response Analyzer EIS300, Warminster, PA, USA). The scan range was ±15 mV relative to corrosion potential (E_corr_). The initial delay was 15 min, and the temperature was 25 °C ± 0.5° C. The polarization resistance (Rp) of the coatings was calculated by the Stern-Geary equation [40], which is based on the fact that a higher Rp value (in Ω cm^2^) corresponds to higher corrosion resistance and to lower corrosion current (i_cor_) as well, i.e., *Rp* ~ 1/i_cor_. The results obtained with this method are highly relevant to standard measurements conducted in natural corrosion conditions. In order to check the reproducibility, the PDP and Rp measurements were repeated at least 3 times.

## 3. Results

Conducting the research presented in this work, we were guided by the following considerations. During the immersion processing of aluminum, the deposited thin CCOC are characterized by cracks and pores that may reach the Al substrate [41]. Their formation is mainly associated with the occurrence of processes of dehydration of CCOC [20]. It is considered that these areas are one of the main reasons that the corrosion protection of CCOC does not always satisfy enough. Such cases eventually require additional post-treatment operations of CCOC [31,42]. In the present study, we applied post-treatment in mixed 0.5 M NaH_2_PO_4_ + 0.1 M Ca(NO_3_)_2_ solution, because during this treatment, according to [37,38] and our XRD and XPS investigations, the following reactions may occur:

2Al + 6NaH_2_PO_4_ + 2Ca(NO_3_)_2_ + 14H_2_O → 2CaHPO_4_(H_2_O)_2_ + 2Na_3_Al(OH)(HPO_4_)(PO_4_) + 4NH_4_OH + 8O_2_ + 3H_2_ (1)

The products obtained during Reaction (1) Ca(HPO_4_)(H_2_O)_2_) and (Na_3_Al(OH)(HPO_4_)(PO_4_) can interact with water according to the following equation [39]:

CaHPO_4_(H_2_O)_2_ + Na_3_Al(OH)(HPO_4_)(PO_4_) + 5H_2_O → Al(OH)_3_ + 3NaOH + Ca(OH)_2_ + 3H_3_PO_4_ (2)

in which the formed Al(OH)_3_ (characterized and proven by XRD analyses [39]) is mainly in the form of its thermodynamically stable phase bayerite–α-Al(OH)_3_.

Along with that, however, the H_3_PO_4_ formed in Reaction (2) can cause Reaction (3) to occur as well [43]

10Ca(OH)_2_ + 6H_3_PO_4_ → Ca_10_(PO_4_)_6_(OH)_2_ + 18H_2_O (3)

which will lead to the deposition of the practically water-insoluble hydroxylapatite (Ca_5_(PO_4_)_3_(OH)) (labeled also as calcium hydroxylapatite (Ca_10_(PO_4_)_6_(OH)_2_) on the S2 system (including on the “bared” areas of the Al substrate—the bottoms of the cracks and pores of the CCOC reaching the Al surface).

We also consider that H_3_PO_4_ formed during Reaction (2) can interact both with the dense regions of CCOC (made up of a mixture of easily soluble Ce_2_O_3_ and hardly soluble CeO_2_ [32,35,42]), as well as with the exposed Al surface (in the areas of microcracks and pores), according to Reactions (4) and (5).

Ce_2_O_3_ + 2 H_3_PO_4_ → 2CePO_4_ + 3H_2_ (4)

2Al + 2 H_3_PO_4_ → 2AlPO_4_ + 3H_2._ (5)

which will lead to the formation of the water-insoluble CePO_4_ and AlPO_4_ on these surfaces. Confirming this surmise are the XPS studies conducted and presented below on the chemical state of the elements Ce, Al, P and Ca, proving the presence of CePO_4_, AlPO_4_ and Ca_5_(PO_4_)_3_(OH) on the surface of the examined samples (see Section 3.3. XPS Studies, below).

### 3.1. SEM/EDS Studies

**Figure 1:**
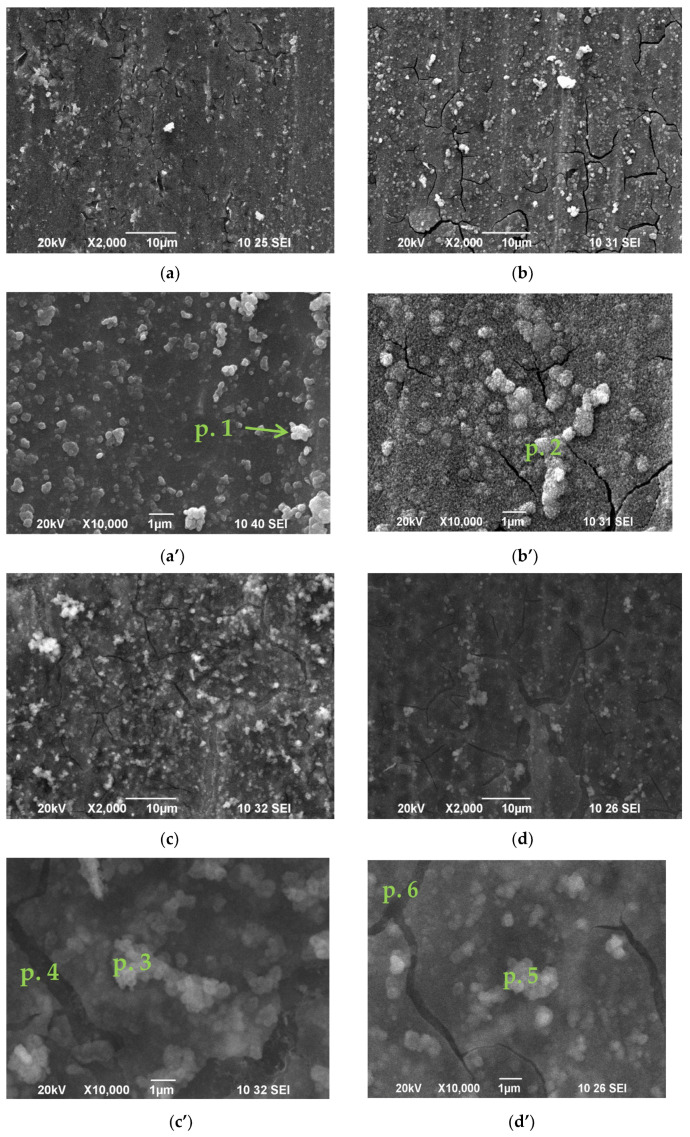
SEM images of studied samples: (**a**) S2; (**b**) S3; (**c**) S4; (**d**) S5 at magnification ×2000; (**a’**) S2; (**b’**) S3; (**c’**) S4. In point 3: Al–27.81%; O–64.56%; P–4.75%; Ca–0.52%; Cu–0; Ce–1.69%; Fe–0.66%. In point 4: Al–90.27%; O–7.66%; P–2.07%; Ca–0%; Cu–0%; Ce–0%; Fe–0%; (**d’**) S5 at magnification ×10,000. In point 5: Al–34.66%; O–34.04%; P–9.37%; Ca–2.13%; Cu–1.03%; Ce–17.88%; Fe–0.89%; In point 6: Al–90.05%; O–8.39%; P–1.56%; Ca–0 %; Cu–0 %; Ce–0 %; Fe–0%.

Figure 1 illustrates SEM investigations of the surface of obtained conversion layers, deposited on Al 1050 (after its pre-treatment-S1), and its immersion treatment in solution(s) of 0.5 M CeCl_3_ × 7H_2_O + 1 × 10^−5^ M CuCl_2_ × 2H_2_O followed by different consecutive sealing treatments in mixed 0.5 M NaH_2_PO_4_ + 0.1 M Ca(NO_3_)_2._ From the presented results, it appears that the formed CCOCs (Figure 1a) on the S1 (the system S2) are built of spherical agglomerates, which are relatively uniformly distributed over the entire surface, reproducing the profile of the Al substrates. Therefore, the roughness and the rigidity determined by the mechanical treatment of the rolled Al 1050 sheet influence in a specific way the formation of the chemically deposited CCOC [35].

Pores and cracks are also observed, their width reaching 0.2–0.4 µm (Figure 1a). The integral EDS analysis (Table 2) registered the presence of Al (86.09%), O (10.40%), Fe (0.37%) and Ce (3.14%). At the same time, the performed EDS analyses at a point (at magnification 300,000×) in the bright areas indicated by arrows (in Figure 1a’) characterizing the areas of CCOC deposited on Al_3_Fe-rich phase agglomerates [35,41] in the aluminum matrix of Al 1050 (Figure 1a’) register the presence of Al (47.25%), O (30.95%), Fe (5.13%) and Ce (16.66%).

Figure 1b presents the changes in the structure and composition of the samples subjected to an additional 5-min immersion treatment of the S2 system in mixed 0.5 M NaH_2_PO_4_ + 0.1 M Ca(NO_3_)_2_ solution (the system S3). The surface morphology of this system is similar to that on Figure 1a. One is to note additionally, the increase in the number and size of the spheroidal agglomerates forming the surface morphology, as well as the changes in the shape and size of the formed micro-cracks, is impressive. At the same time, the integral EDS analysis (Table 2) registered a two-fold increased concentration of Ce (6.55%) and the presence of Ca (0.28%), and the concentrations of Al and O, respectively, were 80.83 and 12.34%. Pronounced to a higher degree are also the sections of the Al_3_Fe phase, on which the conversion layers are predominantly formed (Figure 1b’, marked as Point 2) [34,35,41]. The analyzes, at a point (300,000×) in them, recorded a significantly higher concentration of Ce (26.31%), P (2.88%), Ca (1.51%), Fe (0.80%) and Cu (0.67%) (Table 2) [34,35,41].

Figure 1c characterizes the changes in the structure and composition of samples subjected to three additional 5-min (3 × 5 min) immersion treatment of the S2 system in mixed 0.5 M NaH_2_PO_4_ + 0.1 M Ca(NO_3_)_2_ solution (the system S4). The surface morphology of this system is quite different than that on Figure 1b. The micro-profile of the surface of the samples is more uniform and the number of micro-cracks is reduced (mostly the recorded traces of cracks (shown in Figure 1b) are practically filled with conversion coating—Figure 1c). The results of the integral EDS analysis for this system are presented in Table 2. They indicate that the application of three times longer exposure time in mixed 0.5 M NaH_2_PO_4_ + 0.1 M Ca(NO_3_)_2_ solution causes the inclusion of P in about an eightfold higher concentration (6.48 vs. 0.81%) in such a modified system of conversion coatings. At the same time, phosphates and dihydrogen phosphates (see the XPS studies) are also registered at the bottom of the pores (2.07%), where Ca, Cu and Ce are absent (Figure 1c’). That is, the post-treatment in mixed 0.5 M NaH_2_PO_4_ + 0.1 M Ca(NO_3_)_2_ solution causes direct interaction with the Al substrate in the “defective” areas (cracks), on which, however, CCOC and insoluble calcium-phosphate compounds (see Section 3.2 XRD Studies) are not registered.

Figure 1d characterizes the changes in the structure and composition of samples subjected to three additional 5-min (3 × 5 min) immersion treatments of the S2 system in mixed 0.5 M NaH_2_PO_4_ + 0.1 M Ca(NO_3_)_2_ solution and subsequent exposure for 72 h in 95% humidity atmosphere (the system S5). The surface morphology and micro profile of this system to a certain extent are similar to that on Figure 1c. The same is seen for the results of the integral EDS analysis of its chemical composition, but nevertheless, they register certain differences when comparing the results obtained in the EDS analyses at a point. While the surface concentrations of the aggregates forming the conversion coating (Figure 1d’) are Ce–17.88%, Ca–2.13% and P–9.37%, for the system presented in Figure 1c’, these concentrations are significantly lower—respectively, Ce–1.69%, Ca–0.52% and P–4.75%. This significant difference in the concentration of Ce, Ca and P, determined by the exposure for 72 h in 95% humidity atmosphere, is most likely related to the occurrence of Reactions (1)–(4), which lead to the formation on the surface and in the cracks of CCOC of the practically insoluble in the corrosion medium Ca_5_(PO_4_)_3_(OH), CePO_4_ and AlPO_4_. The XPS analyses on the surface of the investigated samples carried out in the present study (see P. 3.3. XPS Studies, below), as well as those registered for similar systems earlier [42], give reasons to assume that to the favorable protective effect of the above-mentioned phosphate compounds can be added the effect due to the formed Al_2_O_3_ and AlOOH [42].

The quantitative characterization of the concentration of the characteristic elements (Ce, Ca, P) found in the volume of the formed conversion layers is presented in Table 2. According to these data, their arrangement in descending order, for each of the studied systems (or their concentration in the chemical compounds formed by them), is the following:

**Ce**: S3 > S2 > S4 > S5;

**Ca:** S4 > S5 > S3;

**P:** S4 > S5 > S3.

These results indicate that when the exposure time of the system S2 in mixed 0.5 M NaH_2_PO_4_ + 0.1 M Ca(NO_3_)_2_ increases, the concentration of Ce decreases. At the same time, the concentration of Ca and P rises. The concentration of Ca and P at 3 × 5 min exposure in the solution is the highest.

### 3.2. XRD Studies

**Figure 2:**
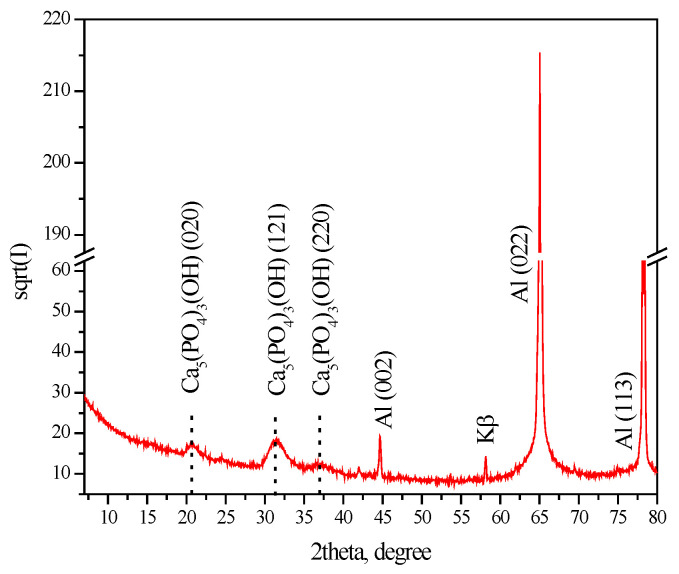
XRD pattern of the system S5 (showed the best corrosion-protective effect) at 2θ = 10°–90°.

The deposited and registered phases on the S5 system (see Section 3.4, below) are shown in Figure 2. Based on the results of the polarization investigations and Rp tests, this sample is characterized by the highest anticorrosion characteristics. It can be seen from the figure that, along with the most salient and prominent peaks (in 2θ scale) of the Al substrate (ICDD PDF #01-073-2661), peaks for the Ca_5_(PO_4_)_3_(OH) (ICDD PDF # 01-070-0798) phase are clearly registered [44,45], while those for the α-Al(OH)_3_ phase are not observed. The processing of the XPS data (see Section 3.3, below), however, indicates the presence of Al(OH)_3_. These results are also in good agreement with the results obtained by EDS analysis of the same samples (see Section 3.1, above), characterizing the presence of elements characteristic for Ca_5_(PO_4_)_3_(OH)—Ca and P (Table 2). These results correspond and confirm the possibility of Reactions (1)–(3), with respect to the formation of Ca_5_(PO_4_)_3_(OH) and Al(OH)_3_, which are difficult to dissolve in the corrosion medium. The latter can inhibit the corrosion process, respectively, its speed, both in the areas of exposed Al substrate (filling out the defects of the CCOC), and thanks to thickening and stabilization of the CCOC, caused by the formation of water-insoluble CePO_4_ and/or AlPO_4_.

### 3.3. XPS Studies

#### 3.3.1. XPS of As-Deposited Samples

Surface XPS studies of the above-described conversion coating systems formed on the aluminum substrate were performed. The surface of the obtained layers was examined both immediately after receiving the samples and after they were subjected to various electrochemical and corrosion tests. The purpose of these studies was to establish the chemical composition of the deposited conversion coatings, the chemical state of the characteristic elements in them before and after being subjected to corrosion attack in CM.

**Figure 3:**
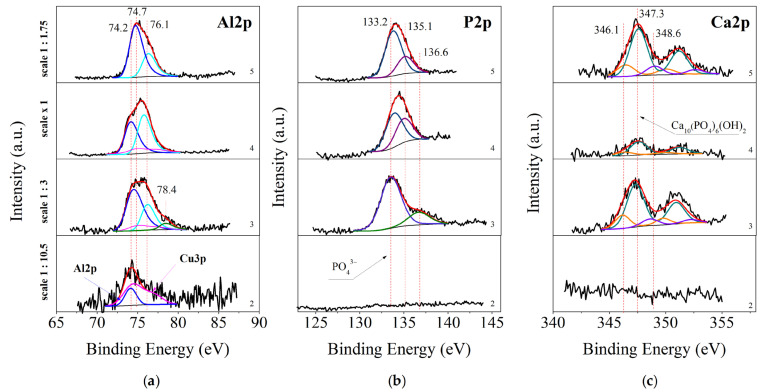
Peak fitting of Al2p (**a**), P2p (**b**) and Ca2p (**c**) XPS spectra for as-deposited samples. From bottom to top: 2—S2, 3—S3, 4—S4 and 5—S5. The sum of peaks is given with a red line, the black line labels the experimental data with the background, and the other color lines represent peak fitting curves.

Figure 3a–c shows XPS–core photoelectron spectra of Al2p, P2p and Ca2p of the as-deposited samples. C1s, O1s, Ce3d, Na1s, N1s and Cu2p spectra are not shown here because they do not provide such essential information about the processes taking place on the surface. The Al2p spectra are shown in Figure 3a. The spectra are deconvoluted into two essential groups of peaks. The first one for samples S2 and S4 at 74.2 eV is attributed to Al_2_O_3_ [46]. This peak for sample S3 is slightly shifted at 74.5 eV to higher BE. Such a shift we can attribute to the presence of AlPO_4_ [47] formed during the immersion treatment. The peak at 74.7 eV for sample S4 is related to the existence of Al(OH)_3_ [48]. The second group around 76.1 eV we can attribute to the presence of some forms of amorphous AlO(OH) with a non-stoichiometric amount of crystalline H_2_O groups [49,50].

In Figure 3b are shown XPS spectra of the other chemical element in the conversion coating–P2p for as-deposited samples. The spectra for sample S3 were deconvoluted into two peaks—at 133.2 eV and around 136.6 eV. This peak at 133.2 eV is a characteristic peak for the presence of PO_4_^3−^ groups [51]. These groups can originate from the phosphate precursor or can be CePO_4_ or AlPO_4_ obtained during the immersion processing (Equations (4) and (5)).

Taking into account the concentration of Na–ions, we can conclude that where the concentration of sodium is higher (Table 3), in sample S4 (134.0 eV) [52], this shift is larger compared to the S5 sample, where this shift is smaller (133.8 eV). From this we can conclude that the amount of unbound phosphate groups in sample S4 is greater than in sample S5. Because NaH_2_PO_4_ is soluble, this may also be a reason for the lower corrosion resistance of this sample, comparable to that of uncoated aluminum. Also a reason for this lower resistance is the lack of a cerium-oxide layer on the surface. In the XPS analysis, this element was not detected. Looking at the P2p spectrum of the sample S3 and comparing it with the chronoamperometric curves, we can note its higher corrosion resistance. Here, the position of the P2p peak is characteristic of the presence of PO_4_^3−^ groups, while approximately 4 at.% Ce^3+^ have been calculated on the surface. Kozhukharov and co-authors assert that when Ce^3+^ is involved in the immersion deposition, CePO_4_ is formed on the Al surface [29]. Considering the areas of Al and P peaks corresponding to Al-PO_4_ bond and P-O bonds (within phosphate groups), the ratio between them should be 1:1, which is the stoichiometry in AlPO_4_. The established ratio, however, is 1:1.5, an excess of P. From here we can conclude that about 33% of the phosphate groups are connected to cerium ions in the form of insoluble CePO_4_. However, the relatively low concentration of cerium phosphate formed is not able to sufficiently block the corrosion centers. The samples S5 and S2 have the highest corrosion resistance. In sample S5, NaH_2_PO_4_ was also registered. Despite the good solubility of NaH_2_PO_4_, the increased corrosion resistance of this sample can be explained by the protective role of Al_2_O_3_ and AlOOH, as well as with the presence of the poorly soluble AlPO_4_ (Table 3). We deduce the presence of AlPO_4_, not the presence of NaH_2_PO_4_, from the smaller shift (133.8 eV) of the phosphate groups to higher binding energy values. In sample S2, on which no phosphate coating was deposited, the relatively good corrosion protection ability can be related to the presence of both Ce_2_O_3_ and Al_2_O_3_.

Figure 3c shows the deconvoluted core photoelectron spectra of Ca2p. They are separated into three doublets. The first peak of Ca2p_3/2_, positioned at 346.1 eV, corresponds to Ca(OH)_2_ [53], which is a product of the chemical interactions taking place on the surface (reaction 2). The second peak, positioned at 347.3 eV, corresponds to the presence of insoluble corrosion-protective Ca_10_PO_46_(OH)_2_ [54,55], also a product of reaction (3), and the third one-to Ca(NO_3_)_2_ [56], which is an element of the precursor system.

The analysis of the Al2p, P2p and Ca2p spectra for the as-deposited samples are confirmed by the peak fitting of the O1s spectra for the same samples (spectra not given in the publication). The spectra can be divided into four groups of peaks. The first group of peaks, positioned at 529.6–531 eV, we can associate to the presence of chemical bounds of oxygen with metal ions. It can be noted that in the sample order S2 (positioned at 529.6 eV) → S3 (530.4 eV) → S4 (530.8 eV) → S5 (531.0 eV), the binding energy of these peaks gradually increases, which we can associate with the decrease of the concentration of cerium oxide and the increase of the concentration of aluminum oxide [46] on the surface. The second group of peaks positioned at 531.4 are related to the presence of P–O–P–bonds within phosphate groups [51]. The third group, peaks shifted relative to it by about one electronvolt, at 532.2–532.6 eV, we can associate with the presence of NaH_2_PO_4_ and OH^−^ groups. The fourth group of peaks, at 535.3–535.5 eV, can be associated with the presence of OOH groups and adsorbed water [49,50].

#### 3.3.2. XPS of Studied Samples after Their Long-Term Exposure in CM

**Figure 4:**
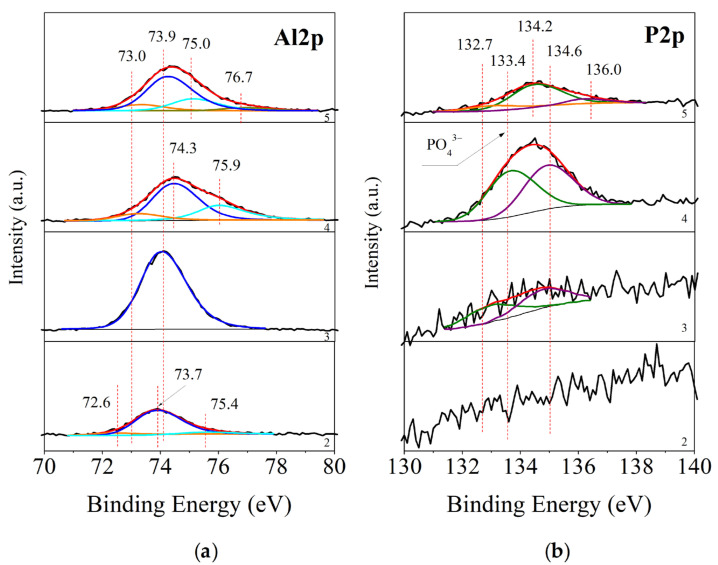
XPS curve peak fitting of Al2p (**a**) and P2p (**b**)–spectra for samples processed in model corrosion medium. From bottom to top: 2—S2, 3—S3, 4—S4 and 5—S5. With a red line is marked the sum of the peaks, the black line labels the originate spectra and the background, and other color lines represent peak fitting curves.

In Figure 4 are presented the results of the XPS analyses of samples identical to the ones discussed above. These samples are studied after long-term exposure in conditions of Rp, OCP and Chronoamperometry tests. The XPS analyses here give us the quantitative and qualitative changes in the chemical composition of the mixed protective conversion layers after these corrosion tests.

##### XPS of Studied Samples after Rp Tests

Figure 4a,b show the deconvoluted spectra of Al2p and P2p for samples S2–S5. In contrast to the results for the as-deposited layers (Figure 3), for sample S4, there is a low intensity peak at 72.6 eV, corresponding to the presence of metal aluminum [57]. Calculating the peak area, we determined that 8.6% of the surface has been completely exposed by the corrosion test, which also accounted for the worse results in (Section 3.4.4. Investigations of Rp, Figure 8) of this sample after the 48th h. The following peak at 73.9 eV corresponds to Al_2_O_3_ [58]. For sample S3, the surface is characterized almost entirely by aluminum oxide. The aluminum peaks for samples S4 and S5 are positioned at 74.1 (sample S5)–74.3 eV (sample S4). A binding energy of 74.1 eV is typical for Al_2_O_3_, and its shift to higher values of 74.3 eV in sample S4 is a sign of the formation of Al-PO_4_ bonds [59]. In the deconvolution of the Al2p peak of samples S4 and S5, another group of peaks is formed at 75.0 eV (sample S5), characteristic of Al(OH)_3_, and at 75.9 eV [60], characteristic of amorphous non-stoichiometric AlO(OH) + H_2_O.

By comparing the P2p spectra for these samples, they can be separated into two peaks. For the S3 sample, they are located at binding energies of 132.7 and 134.6 eV. Both peaks are with a low-intensity, which corresponds to the low phosphorus concentration of 0.7 at.% on the surface after the corrosion test. The peak at 132.7 eV can be associated with the presence of phosphorus within the Na_2_HPO_4_–compound [61], and the peak at 134.6 eV corresponds to Na–P–O–bonds in NaPO_3_ [62], which also corresponds to the low concentrations of 0.4 at% of Na found in the analysis. The presence of a certain amount of AlPO_4_ is also possible. Therefore, oxidation-reduction processes of conversion of the phosphorus-containing component of the precursor into its more soluble compounds took place on the surface of this sample.

The concentration of phosphorus on the surface of sample S4 is the highest compared to the other samples subjected to Rp tests–4.4 at. % (Section 3.4.4. Investigations of Rp, Figure 8). The photoelectron peak is split again into two peaks, at 133.4 and 134.7 eV. The first peak positioned at 133.4 eV can be associated with the presence of phosphate groups. The second peak can be associated with the presence of PO_3_ groups, probably AlPO_3_. The phosphorus concentration for sample S5 is lower–3.5 at.%. The spectrum of P2p is divided into three peaks–132.7, 134.2 and 136.0 eV. The first of them corresponds to the presence of hydrogen phosphate groups, the second to -PO_3_ bonds [63] and the third is probably due to poorly adsorbed phosphate groups dispersed on the Al surface.

Here again, the analysis of the spectra of the Al2p, P2p and Ca2p Rp samples are confirmed by the peak fitting of the O1s spectra for the same samples (spectra not given in the publication). Here, the O1s spectra can be divided into three groups of peaks. The first group, at 530.1 eV, corresponds to the presence of metal oxides [46]. The second, at 530.7 eV, we can associate to the presence of a mixture of phosphates and hydrogen phosphates [51]. The third group, at 532.8 eV, we can associate with the presence of OH^−^ and OOH^−^ groups [49,50].

Analyzing the spectra and concentrations of the chemical elements on the surface, we can conclude that in the Rp tests, the best resistance will be shown by the layers with the presence of phosphate groups present on the surface. Sample S4, which has the highest concentration of phosphate groups (4.4 at. %), however, does not exhibit the best corrosion resistance, which also agrees well with the chronoamperometric studies. For sample S4, the presence of the more soluble AlPO_3_ and the absence of a cerium conversion layer are combined. In this regard, it can be assumed that the most important role for the protective ability is played by Al_2_O_3_, Al(OH)_3_, and amorphous AlO(OH) + H_2_O, respectively, in samples S3 and S5. It should be noted that after the corrosion tests of the S2 sample, 2.4 at. % Ce_2_O_3_ remained on its surface, which from the Ce3d spectrum analysis shows to be in the form of Ce^3+^, the more soluble form of cerium phosphate [64]; it is, therefore, washed away after 48 h.

##### XPS of Studied Samples after OCP Tests

XPS-surface studies of samples subjected to corrosion tests at OCP were performed. The spectra of Al2p spectra can be divided into three groups of peaks—at 72.9 eV, 73.7–74.6 eV and 76.1 eV (they are not included in this paper). They correspond to the presence of metallic aluminum [65], a mixture of Al_2_O_3_ and Al(PO)_4_ and AlOOH [49,50]. In the Al2p-spectrum of the S2 sample, no peak associated with the presence of pure metallic aluminum is observed, indicating that the surface of this sample was not exposed as a result of the corrosion test. The largest amount of Ce was also found on the surface of this sample—5.7 at.%. In this case, it is in the form of Ce^3+^. In the Al2p spectra of samples S4 and S5, the peaks associated with the presence of metallic aluminum are with a low intensity. They are with the areas corresponding to 13–13.7% of the total amount of Al. This fact indicates that in these samples, this surface baring as a result of the corrosion tests was minimal. In the S2 sample, the first of the second group of peaks is positioned at 73.7 eV. The lower binding energy compared to that of Al_2_O_3_ (74.1 eV) and the high concentration of Ce on the surface of this sample may be an indication of the formation of a mixture of Al_2_O_3_ and CeAlO_3_ [66]. In samples S4 and S5, the binding energy of the peaks is 74.4–74.6 eV, typical of aluminum phosphate. The ratio between Al and phosphorus is 1.8:1. The stoichiometric ratio of Al to P in AlPO_4_ is 1:1. This excludes the possibility the entire amount of Al being covered with phosphates. For this reason, the peaks at 74.4–74.6 eV in the Al2p spectrum can be attributed to a mixture of Al_2_O_3_ and AlPO_4_, although the binding energy is typical only for AlPO_4_.

The P2p spectra for the studied samples can be divided into two peaks positioned at 133.3 and 137.0 eV. The first peak, already mentioned above in the text, corresponds to the presence of P–O–bonds within aluminum phosphates. The second peak we can again associate with the presence of phosphate agglomerates located on the surface and accordingly, with a different charge.

The Ca established on the surface of samples S4 and S5 Ca is in minimal concentrations—0.2–0.6 at.%. Despite the noisy spectra, their deconvolution was possible and for sample S4, the doublet was split into a single peak positioned at 347.3 eV. This peak corresponds to Ca_10_PO_46_(OH)_2_ [54,55]. For sample S5, its spectrum can be divided into three peaks, the maxima of which are at 346.3, 347.3 and 348.5 eV. Just as with the as-deposited samples, they correspond to the presence of Ca(OH)_2_, a product of chemical reactions occurring on the surface (reaction (2)), insoluble Ca_10_PO_46_(OH)_2_ [54,55], also a product of Reaction (3) and Ca(NO_3_)_2_, which was part of the precursors. The percentage ratio between the three peaks is 31.0:50.3:19.6%. From this we can conclude that half of the calcium is in the form of insoluble Ca_10_PO_46_(OH)_2_.

##### XPS of Studied Samples after Chronoamperometric Tests

The XPS spectra of the samples characterized by stable corrosion behavior after 168 h of exposure in CM (see Section 3.4.3. Chronoamperometric Investigations) were taken—samples S2 and S3 (the spectra are not given in this work). Only aluminum and oxygen were found on their surface. The Al2p spectra of the two samples can be divided into three main groups of peaks—72.7 (sample S2), 73.2 eV (sample S3), 74.1 eV and 75.3 eV. The peak positioned at a binding energy of 72.7 eV for the S3 sample is typical for the presence of metallic aluminum [57]. Its area represents only 6% of the total amount of aluminum, i.e., only 6% of the surface is bared in the chronoamperometric test. For the S3 sample, the peak with the lowest binding energy is positioned at 73.0 eV. This energy is 0.3 eV higher than typical for metallic aluminum, and therefore, we can associate it with the presence of slightly oxidized aluminum. In this sample, the peak is more intense and represents 11.4% of the total area, or of the concentration of aluminum. The peak positioned at 74.1 eV, present in both samples, is typical for the presence of Al_2_O_3_ [46]. For the S2 sample, it is 78% of the total area, and for the S3 sample, it is 73 % of the total area of aluminum. The peak positioned at 75.3 eV, again present in both samples, is characteristic of the presence of AlOOH [42].

### 3.4. Electrochemical Investigations

#### 3.4.1. Polarization Curves

**Figure 5:**
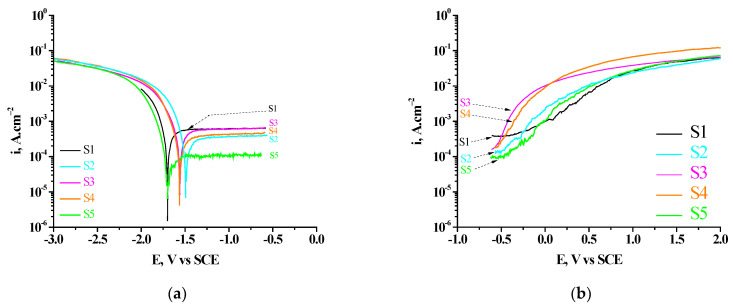
Potentiodynamic polarization curves of the studied systems: S1; S2; S3; S4 and S5 in cathode (**a**) and anodic (**b**) directions.

The anodic and cathode potentiodynamic polarization curves of the studied systems are presented in Figure 5. As was marked above (see in paragraph 2), these measurements were started in cathode and anodic directions from the E_OCP_ of S1. They characterize the electrochemical behavior of the S1 (bar) substrate as well as consecutive obtained conversion coatings formed on it in model corrosion medium 0.1 M NaCl. On Figure 5a and Table 4, respectively, it is seen that the polarization of the studied samples in the cathodic direction causes the formation of a wide range of potentials (~1000 mV), in which the value of the current of passivity does not depend on the change in the value of the potential. This effect illustrates and characterizes this interval of potentials as passive layer area (E_PLA_), after which started the processes of breakdown of the passive layers. At the same time, depending on the type and duration of the immersion post-treatment of the samples in PhCa, a shift of E_corr_ of the studied samples in a positive direction is observed. This shift is most pronounced (by ~200 mV, compared to E_corr_ of S1 substrate) for the system S2. It is less—by ~ 160 mV—for the systems S3 and S4, while for the system S5, E_corr_ is ~25 mV more positive than E_corr_ of S1. At the same time, the decrease of i_corr_ for the studied systems follows the relationship: i_corr_ of S1 > i_corr_ of S2 > i_corr_ of S3 > i_corr_ of S4 > i_corr_ of S5–Table 4.

The comparison of the obtained polarization curves establishes a significant change in the width of the passive state zone (E_r.p.s._, V), as well as in the current (at the plateau) of the passive state (i_pass_, A.cm^−2^) of the studied systems. From the data systematized in Table 4, it can be seen that: (1) the most positive value of E_corr_ for the studied samples is observed for the system S2 (−1.472 V); (2) the lowest value of the corrosion current i_corr_ (1.7 × 10^−5^ A.cm^−2^) was registered for system S5; (3) the best corrosion indicators are established for system S5 both in terms of the lowest value of the current of passive state (i_pass_) (1.0 × 10^−4^ A.cm^−2^) and the widest range of passive state (E_rps_) (−1.537 ÷ −0.470 V)—Table 4.

The registered specific course of the polarization curves (Figure 5a), in the region from potentials −0.680 V to −1.705 V, characterizes and describes the changes in the anodic behavior of the aluminum substrate due to its modification with effective cathodic coatings. The presence of a well-defined, wide passive region in the course of the obtained polarization curves is observed, falling in the region of cathodic potentials characteristic of the modifying cerium oxide layers. These results are related to the following phenomenon: according to the data from a previous study [32,33,34,35] and the present one (see Section 3.1, Section 3.2 and Section 3.3), both the S1 substrate and the conversion coatings formed on it are characterized by thin surface layers that differ in composition and structure. The potentiodynamic unfolding of the potential (1 mV.s^−1^) in the interval from −0.660 V to −1.5 V for the different systems, although in a negative direction, will lead to partial or complete dissolution of ceria in components of the deposited conversion layers. This is due to the fact that the E_corr_ of the S1 substrate is more electronegative than the E_corr_ of the studied protective, conversion systems. That is, the area more positive than −1.705 V is an anodic region for the deposited (on S1) protective layers, in which their dissolution is possible after reaching: −1.682 V for system S5; −1.553 V for system S4; −1.550 V for the S3 system; −1.472 V for the S2 system.

The results presented above correspond to the anodic polarization curves obtained for the same samples-Figure 5b. On Figure 5b and Table 4, respectively, it can be seen that the polarization of the studied samples (beginning from E_OCP_) in the anodic direction maintains the passive state of the S1 substrate up to ~−0.350 V (curve 1); of the S2 system (curve 2)—up to ~−0.480 V; of the system S3 (curve 3)—up to ~−0.585 V; of the system S4 (curve 4)—to ~−0.593 V, and for the system S5 (curve 5)—up to ~−0.470 V. After reaching these values, the course of the anodic polarization curves (in the interval ~−0.500 V–0 V) is characterized as a transition to overpassivation of the samples (transpassive region), and after its further increase (in the interval ~ 0 V–+0.250 V), this stage of the polarization curves more probably is connected with the passive layer breakdown [67].

Briefly, we are led to conclude that this part of cathode curves (started in negative direction from the E_OCP_-after reach of E_corr_, respectively) for all of the studied samples correspond to the process of reduction of oxygen. At the same time (as it is shown above), the part of cathode curves obtained in the area of potentials E_r.p.s._-E_OCP_ and the anode curves (obtained in positive direction, after E_OCP_ of the studied samples) correspond to the process of passivation. They contain all typical steps (local corrosion due to passive layer breakdown; region of passive state; transpassive zone; area of oxygen evolution) which are representative for the all investigated samples. These results, obtained under conditions of external potentiodynamic polarization inform about the kinetics of the related corrosion process reactions, but do not characterize them fully since the real corrosion processes occur at the open circuit potential (OCP). At the same time, it is known that the change in OCP (E_OCP_) as a function of immersion time can be used to monitor the chemical stability and corrosion process of the Al alloys [68]. Although E_OCP_ does not provide any direct information on the corrosion kinetics, it suggests the corrosion susceptibility [69].

This is the reason why when organizing our experimental studies, along with these considerations, we considered the possibility of the formation of a set of phosphate complexes (obtained at different PhCa-post-treatments of the CCOC layers) with different solubility [36,37,38,39] during corrosion attack of Cl^−^. In connection with this, our research was conducted both immediately after receiving (as-deposited) the studied systems and after 168 h of their exposure in the CM.

To gain the needed additional insights, we conducted investigations of self-occurring corrosion processes in the systems of interest at: the change in OCP values monitored E_OCP_ vs. time plot; the changes in anodic current (i_a_) transients at pitting potential (E_pit_); the changes of polarization resistance (Rp) as a function of samples exposure time in CM (in OCP conditions).

#### 3.4.2. E_ocp_ vs. Time Plotcharacterization

**Figure 6:**
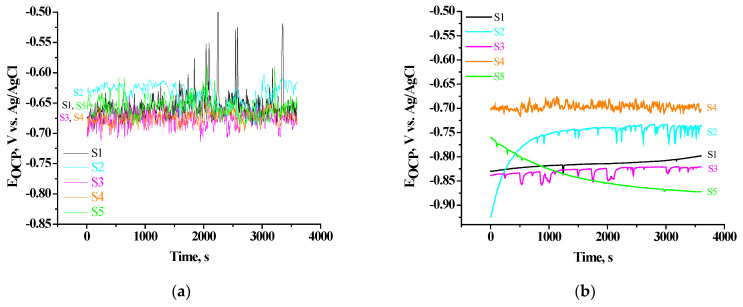
OCP vs. time plots during immersion in a 0.1 M NaCl solution for as-deposited samples (**a**) and after 168 h exposure of the same samples in CM (**b**): Bare S1 substrate; S2; S3; S4; S5.

As it is known, the change in OCP as a function of the immersion time can be used to monitor the chemical stability and corrosion process of the studied samples, i.e., to assess their corrosion susceptibility. In this relation and in light of the marked above, we realized systematically investigations of the studied systems illustrating the change of their OCP vs.time in CM. Figure 6 shows the results obtained for the studied as-deposited (Figure 6a) samples and samples identical of them after 168 h of their exposure in the CM (Figure 6b). It was established on Figure 6a that during the immersion for 3600 s, the course and the changes in E_OCP_ for the bare (S1) sample and system S5 practically coincide at ~−0.660 V. For the systems S3 and S4, both coincide at ~–0.680 V. While for the system S2, this change of E_OCP_ goes in a positive direction till ~−0.630 V. The big fluctuations observed for practically all of the studied systems point to dissimilarities in the potential of different intermetallic inclusions, metastable pit formation and re-passivation in the CM. The fluctuation in the OCP could be due as well to the partial incorporation of the aqua ion and formation of Al oxides/hydroxides. When the samples are exposed to the aggressive medium, the corrosive ions penetrate through the active regions and lead to a more negative OCP. In total, the average positive change of E_OCP_ is at its maximum under the action of ceria conversion treatment (S2) is of ~0.030 V. This positive shift (in the range of E_OCP_ from ~−0.660 to ~–0.630 V) compared to the S1 substrate indicates the effective incorporation and also the stability of the ceria-modified Al substrate. Obviously, at the as-deposited systems S3, S4 and S5, the maximum protective effect is not reached, the maximum positive change of E_OCP_, respectively, characteristic for the system S2.

The analogous measurements realized after exposure of the as-deposited samples in the CM for 168 h are shown in Figure 6b. It is seen from the course of changes of the E_OCP_ that the tendency of the change of E_OCP_ in this case is quite different. (1) All E_OCP_ transients are shifted from the area −0.680–0.630 V to the area −0.840–0.700 V. At the same time, relative to the E_OCP_ of the Al substrate (E_OCP_ of S1 ~ −0.815 V), with the most positive value for E_OCP_ characterizing system S4 (E_OCP_ ~ −0.700 V), followed by system S2 (E_OCP_ ~ 0.760 V), while systems S3 and S5 are characterized by more negative values for E_OCP_ (−0.830 and −0.840 V, respectively). These results are in good agreement with the data from EDS and XPS studies for the same samples, presented in Table 2 and Table 3. From the results, therein, it can be seen that there is a direct relationship between the disposition of E_OCP_ transients (Figure 6) and correspondingly determined values for volume (Table 2) and surface (Table 3) concentrations of the main characteristic elements (Ce, P, Ca) of the formed conversion layers. From this, a conclusion can be drawn that the corrosion-protective ability (of these essentially cathodic coatings) in the as-deposited samples is dominated by the CCOC layers. While after prolonged exposure (168 h) in CM, the corrosion protection of S1 is mainly based on the layers formed after the PhCa-sealing post-treatment of system S2. According to the results of the XPS analyses, these are mainly the additionally formed agglomerates and/or layers of: Ca_5_(PO_4_)_3_(OH), CePO_4_ and AlPO_4_ and AlOOH and Al(OH)_3_, as given by reactions 1–5. Complementing these results and conclusions, the above-mentioned (Section 3.4.1. Polarization Curves) conclusions can be added that the manifestation of zones of passive state (Figure 5), before reaching the values of the registered E_corr_ of the delayed conversion coatings, is completely natural. They indicate the change in the character of the corrosion process, as a result of the movement of the corrosion potentials from the zone of active dissolution to the zone of passivity of the aluminum substrate.

#### 3.4.3. Chronoamperometric Investigations

**Figure 7:**
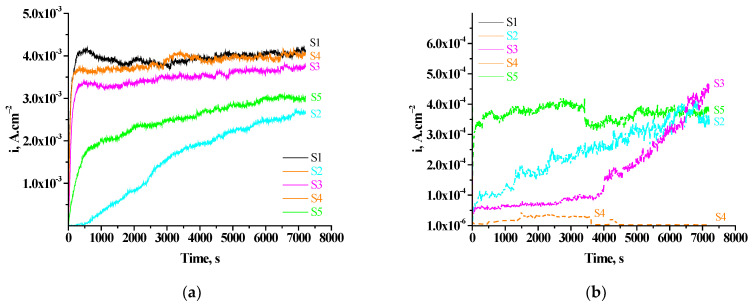
Chronoamperometric i_a_-Time transients: (**a**) for as-deposited samples S1; S2; S3; S4, S5_,_ recorded in 0.1 M NaCl at the pitting potential of Al 1050 (E_pit_ = –0.550 V vs. Ag/AgCl), and (**b**) after exposure of the same samples for 168 h in 0.1 M NaCl and following analogous chronoamperometric investigation during 7200 s.

In these investigations, by polarizing the samples anodically at E_pit_, we aimed to approach to a maximal extent the actual corrosion process, respectively, to characterize corrosion in view of pitting corrosion, which is a basic characteristic of aluminum and its alloys in Cl^−^-containing CM [26,70]. Based on the course of the registered curves, we could judge the character of the corrosion attack and the appearance of pitting damages. Figure 7 presents the results for the studied samples.

As it is observed in Figure 7a, for the S1 (unprotected Al substrate), after its immersion in CM at E_pit_ (–0.550 V vs. Ag/AgCl [70]), the i_a_ density increases sharply (until the ~350-th s of exposure) up to values ~4.22 × 10^−3^ A.cm^−2^, whereupon the surface film on the Al is disrupted (Figure 7a), transient S1), which is a prerequisite for the appearance and development of pitting corrosion during the interaction with the CM. After breaking through the passive film, there starts a process of local corrosion characterized by values of the anodic current (i_a_) ~3.84 × 10^−3^ A.cm^−2^ (at 3600 s) and the appearance of current oscillations specific for it owing to the unstable pits that are repassivated/activated. Similar behavior is also observed for the current transients of the S4 and S3 systems (Figure 7a) for which a process of local corrosion is characterized by very near values of the i_a_ −4.05 × 10^−3^ A.cm^−2^ and 3.51 × 10^−3^ A.cm^−2^, respectively.

The course of the current transients is essentially different for the samples protected by CCOC_(Ce+Cu)_ and sealed in PhCa _(3 × 5 min + 72 h exp.)_ solutions (Figure 7a)—transients 2 and 5, respectively. Current fluctuations characteristic for localized breakdown of the passive film decrease, which would lead to the decrease of initiation and growth of corrosion pits in the CM. The transients have a process course characteristic of general corrosion, the i_a_ of which are 2.54 × 10^−3^ and 1.84 × 10^−3^ A.cm^−2^.(at 3600 s) for system S5 and S2, respectively.

The comparison of the current transients in the case of a consecutive protected by CCOC_(Ce+Cu)_ and sealed at different PhCa regimes of the S1 shows (Figure 7a) that the order of stabilities of the systems towards the appearance and development of pitting corrosion is the following: (S1) < S4< S3< S5 < S2.

At the same time, the transients shown in Figure 7b, which characterize analogous to Figure 7a samples, after their 168 hexposure in CM, reveal quite different results. The current transients are characterized by a very fast establishment (till 10-th-100-rds) of constant values of i_a_, which are an order of magnitude lower (2.59 × 10^−4^–2.72 × 10^−5^ A.cm^−2^) than those obtained for the as-deposited samples (Figure 7b). After the 3600th s of exposure in the corrosion medium, they are characterized by values for i_a_, respectively, for: transient 2–2.59 × 10^−4^ A.cm^−2^; transient 3–9.40 × 10^−4^ A.cm^−2^; transient 4–2.79 × 10^−5^ A.cm^−2^; transient 5–3.19 × 10^−4^ A.cm^−2^. In the interval of times (3600–7200 s) of exposure in CM, the values for i_a_ reached at 3600 s start to rise, reaching ~3–4.5 × 10^−3^ A.cm^−2^ for systems S2, S5 and S3. At system 4, however, a unique decrease in i_a_ is registered, maintaining a constant value (in the interval 3600–7200 s) of 1 × 10^−6^ A.cm^−2^. The explanation of this effect will be the subject of our next study.

#### 3.4.4. Investigations of Rp vs.Time of Exposure in CM

**Figure 8:**
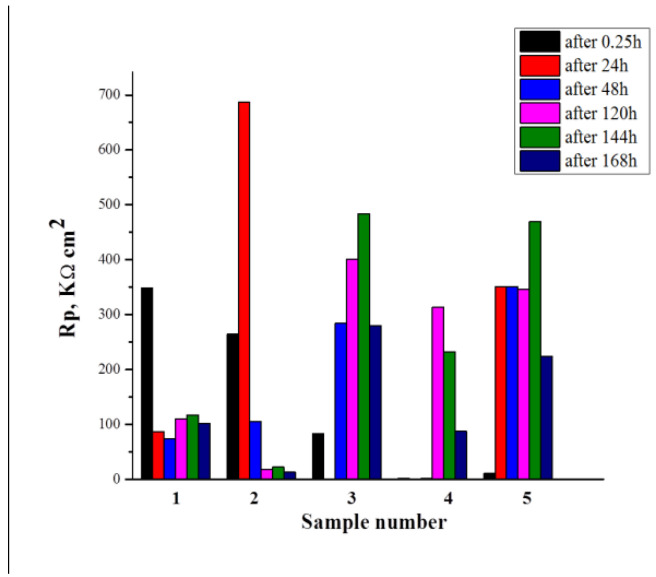
Changes in Rp of the studied systems as a function of the exposure time 0.25 (the initial delay of 15 min before starting of measurements of Rp)—168 h in the CM (1—S1; 2—S2; 3—S3; 4—S4, 5—S5).

Figure 8 shows the results reflecting the changes in Rp of the studied systems as a function of the exposure time (0.25–168 h) in the CM. The analysis of the results obtained showed that after the first hour of exposure of S1 in CM, its polarization resistance (Rp) is characterized by a value of ~350 kΩ.cm^2^. In the subsequent interval of exposure times, 24–168 h, it decreased more than threefold, varying in the interval 75–115 kΩ.cm^2^.

The formation of a CCOC_(Ce+Cu)_ layer on S1 (the S2 system) is characterized by values for Rp~ 700 kΩ.cm^2^, after its exposure in CM for 24 h. At the subsequent exposure times (48–168 h) in CM, however, this value drastically decreases to ~20 kΩ.cm^2^. This effect can be related to the strong decrease in the concentration of Ce_2_O_3_ (15.8% vs. 2.4%—Table 3) due to the dissolution of CCOC, which will lead to the exposure and activation of certain (containing Fe and Cu) areas of the Al substrate [34].

The sealing post-treatment of system S2 in NaH_2_PO_4_ and Ca(NO_3_)_2_ solution, leading to the formation of system S3, causes a significant increase in Rp—up to ~480 kΩ.cm^2^ after the 144th h of exposure in CM. After the 168th h of CM exposure, however, this value drops to ~280 kΩ.cm^2^. This change is most likely due to the strong decrease in volume (Table 2) and surface (Table 3) concentrations of Ce and P after the 168th h of exposure in the CM.

In system S4, characterized by three-fold sealing post-treatment, the values of Rp change are significantly lower (up to ~5 kΩ.cm^2^ after 48th h of exposure), then after the 120th h, they rise to ~310 kΩ.cm^2^, and after the 168th, they decrease again to ~85 kΩ.cm^2^. In this case, the recorded strong decrease in Rp after the 168th h of exposure in the CM is probably related to the strong decrease in bulk (0 wt.% Ce and 0.89 wt.% P-Table 2) and surface (0 at.% Ce, 4.4 at.% P and 3 at.% N-Table 3) concentrations of Ce, P and N after the 168th h of exposure in CM. At the same time, as indicated in the XPS analysis paragraph, in this sample, apart from the absence of cerium oxide and/or phosphate, phosphorus is in the form of the more soluble AlPO_3_.

For system S5, which included an additional 72 h treatment at 50 °C in an atmosphere with 95% relative humidity, however, the values for Rp increase again, reaching and exceeding those for system S3. Moreover, they are higher (up to ~350 kΩ.cm^2^) and unchanged (up to ~144 h of exposure in CM) compared to system S4. The high values registered for Rp after the 168th h of exposure in CM are in good agreement with the results of XPS analyses of the changes in the surface concentration of the elements characteristic of the deposited conversion layers (Table 3). They describe the presence of cerium-phosphate layers, the concentration of the elements in which it is 28.2 at.% Al and 3.5 at.% P (the rest up to 100 at.% is oxygen). In these cerium-phosphate layers, aluminum is in the form of insoluble Al_2_O_3_, Al(OH)_3_, and amorphous AlO(OH) + H_2_O, which determine its relatively high corrosion resistance.

## 4. Conclusions

Ceria-based conversion coatings, formed on technically pure Al-1050, were post-treated by sealing in mixed 0.5 M NaH_2_PO_4_ + 0.1 M Ca(NO_3_)_2_ solution. We paid extra attention to the influence of regimes of this post-treatment operation. Of as-prepared samples, we have characterized the chemical composition in the volume and on the surface by XRD and XPS. The results allow to ascertain the following conclusions:

1. There is substantial influence of the time and type of sealing post-treatment on the chemical composition and chemical state of the elements in the obtained and investigated systems. It is established there is a strong decrease of the concentration of Al_2_O_3_ and Ce_2_O_3_ components in the as-deposited CCOCs at the expense of the formation of insoluble phases: Ca_5_(PO_4_)_3_(OH); AlPO_4_; CePO_4_ and AlOOH (transformed in maximal stage of increase in Al(OH)_3_), as well as PO_3_^−^, compounds with Al and Ce, after their sealing post-treatment in mixed sodium phosphate and calcium nitrate solution;
2. Based on the analyzed specific course of the potentiodynamic polarization dependences, changes in the anodic behavior of the aluminum substrate have been established, which are due to its modification with effective cathodic coatings;
3. The comparison of these results with the changes of the concentrations of Ce^3+^, Al and P (also their respective oxides and phosphates) before and after exposure of the samples in CM show that the concentrations and chemical state of the Al, Ca, Ce and P (and their form as protective compounds) on the surface of the studied samples are directly related. In this case, it was found that the combination of oxide-phosphate compounds forming the conversion layers of system S5 has the best protective effect;
4. Polarization investigations simultaneously showed that the combination of studied phosphate and ceria conversion layers are not only cathodic barrier coatings, but they also change the kinetics of the conjugated electrochemical reactions characterizing the corrosion process in Cl^−^-containing media, i.e., they determine the electrochemical protection of Al substrates.

The established protective effect of the mixed conversion coatings on Al at long exposure in CM can be related to the beneficial transformation of the chemical composition of CCOC, formed on Al substrates after sealing post-treatment processing. This effect, as well as the formation of different types of corrosion products (at a long time of exposure in CM) on the surface of Al/CCOC/PhCa systems provide an effective barrier to the diffusion of Cl^−^ toward to the Al surface, which leads to the corresponding positive and beneficial changes of the i_a_ and Rp for the studied systems.

## Figures and Tables

**Table 1:** Treatment conditions of the studied samples.

Type of Treatment of the Samples			Abbreviation of the Samples
in NaOH (1.5 M; 60 °C; 1 min) Solution	in 0.5 M CeCl_3_ × 7H_2_O + 1 × 10^−5^ M CuCl_2_ × 2H_2_O (25 °C; 2 h)	in 0.5 M NaH_2_PO_4_ + 0.1 M Ca(NO_3_)_2_ Solution (85 °C)	
S1	YES	NO	NO
S2	YES	YES	NO
S3	YES	YES	5 min.
S4	YES	YES	3 × 5 min.
S5	YES	YES	3 × 5 min + 72 h in humidity atmosphere

**Table 2:** Chemical composition of the studied systems determined by EDS analysis before and after their exposure in CM for 168 h.

Sample	Al, wt.%	O, wt.%	Ce, wt.%	Ca, wt.%	P, wt.%	Cu, wt.%	Fe, wt.%	Cl, wt.%
S2 as-deposited	86.09	10.4	3.14				0.37	
after 168 h in 0.1 M NaCl	93.68	4.68	1.64				0	
S3 as-deposited	80.83	12.34	6.55	0.28	0	0	0	
after 168 h in 0.1 M NaCl	88.87	8.67	1.33	0	1.13	0	0	0
S4 as-deposited	63.97	25.57	2.58	0.95	6.48	0.45	0	
after 168 h in 0.1 M NaCl	91.38	7.73	0	0	0.89	0	0	0
S5 as-deposited	68.94	22.64	2.12	0.84	5.1	0	0.35	0.37
after 168 h in 0.1 M NaCl	94.48	5.15	0	0	0	0	0	

**Table 3:** Chemical composition of the elements of the studied systems determined by XPS analysis for the following samples: as-deposited(the first row); after Rp measurements for 168 h (the second row), after 168 h Chronoamperometry (the third row) and after 168 h OCP (the fourth row).

Sample	Al, at.%	O, at.%	Ce, at.%	Ca, at.%	P, at.%	Cu, at.%	Na, at.%	N, at.%
**S2** as-deposited	11.0	70.5	15.8			2.7		
after 168 h Rp	20.5	76.7	2.4			0.4		
after 168 h Chronoamperometry	26.0	74.0						
after 168 h OCP	16.2	78.1	5.7					
**S3** as-deposited	17.1	61.8	3.84	2.3	14.3	0.6		
after 168 h Rp	27.7	71.2			0.7		0.4	
after 168 h Chronoamperometry	30.5	69.5						
**S4** as-deposited	34.7	53.4		0.4	8.8	1.4	0.7	0.6
after 168 h Rp	29.9	62.6			4.4			3.0
after 168 h OCP	24.2	65.2	0.5	0.2	6.8			3.0
**S5** as-deposited	19.6	65.1	0.5	1.7	11.6		0.6	1.0
after 168 h Rp	28.2	68.4			3.5			
after 168 h OCP	24.0	63.4	1.1	0.6	8.2			2.7

**Table 4:** Electrochemical parameters-corrosion potential (E_cor_)_,_ corrosion current (i_cor_)_,_ current of passivity (i_pass_) and range of passive state (E_r.p.s._) of the studied systems, determined on the base of polarization investigations.

Sample	E_cor_, V	i_cor_, A.cm^−2^	i_pass_, A.cm^−2^	E_r.p.s._, V
S1	−1.705	9.8 × 10^−5^	5.8 × 10^−4^	−1.425 ÷ −0.587
S2	−1.472	8.1 × 10^−5^	3.4 × 10^−4^	−1.263 ÷ −0.480
S3	−1.550	5.7 × 10^−5^	5.5 × 10^−4^	−1.360 ÷ −0.585
S4	−1.553	4.5 × 10^−5^	3.6 × 10^−4^	−1.388 ÷ −0.593
S5	−1.682	1.7 × 10^−5^	1.0 × 10^−4^	−1.537 ÷ −0.470

## Data Availability

Not applicable.
